# Analysis of the transcriptome of bovine endometrial cells isolated by laser micro-dissection (1): specific signatures of stromal, glandular and luminal epithelial cells

**DOI:** 10.1186/s12864-021-07712-0

**Published:** 2021-06-18

**Authors:** Wiruntita Chankeaw, Sandra Lignier, Christophe Richard, Theodoros Ntallaris, Mariam Raliou, Yongzhi Guo, Damien Plassard, Claudia Bevilacqua, Olivier Sandra, Göran Andersson, Patrice Humblot, Gilles Charpigny

**Affiliations:** 1 Department of Clinical Sciences, Swedish University of Agricultural Sciences, SLU, PO Box 7054, 750 07 Uppsala, Sweden; 2 Faculty of Veterinary Science, Rajamangala University of Technolgy Srivijaya (RUTS), Thungyai, Nakhon si thammarat, 80240 Thailand; 3 Université Paris-Saclay, UVSQ, INRAE, BREED, 78350 Jouy-en-Josas, France; 4 GenomEast Platform CERBM GIE, IGBMC, 67404 Illkirch, Cedex France; 5 Université Paris-Saclay, INRAE, AgroParisTech, GABI, 78350 Jouy en Josas, France; 6 Department of Animal Breeding and Genetics, Molecular Genetics, Swedish University of Agricultural Sciences, SLU, PO Box 7023, 750 07 Uppsala, Sweden

**Keywords:** Cow, Endometrium, Cell type-specific, Transcriptome, LCM, RNA-seq

## Abstract

**Background:**

A number of studies have examined mRNA expression profiles of bovine endometrium at estrus and around the peri-implantation period of pregnancy. However, to date, these studies have been performed on the whole endometrium which is a complex tissue. Consequently, the knowledge of cell-specific gene expression, when analysis performed with whole endometrium, is still weak and obviously limits the relevance of the results of gene expression studies. Thus, the aim of this study was to characterize specific transcriptome of the three main cell-types of the bovine endometrium at day-15 of the estrus cycle.

**Results:**

In the RNA-Seq analysis, the number of expressed genes detected over 10 transcripts per million was 6622, 7814 and 8242 for LE, GE and ST respectively. ST expressed exclusively 1236 genes while only 551 transcripts were specific to the GE and 330 specific to LE. For ST, over-represented biological processes included many regulation processes and response to stimulus, cell communication and cell adhesion, extracellular matrix organization as well as developmental process. For GE, cilium organization, cilium movement, protein localization to cilium and microtubule-based process were the only four main biological processes enriched. For LE, over-represented biological processes were enzyme linked receptor protein signaling pathway, cell-substrate adhesion and circulatory system process.

**Conclusion:**

The data show that each endometrial cell-type has a distinct molecular signature and provide a significantly improved overview on the biological process supported by specific cell-types. The most interesting result is that stromal cells express more genes than the two epithelial types and are associated with a greater number of pathways and ontology terms.

**Supplementary Information:**

The online version contains supplementary material available at 10.1186/s12864-021-07712-0.

## Background

In all mammals, successful establishment of pregnancy depends on timely interactions between the developing embryo, the uterine milieu and the endometrium. In species of economic interest such as cattle, a large number of studies have been conducted to establish the basis of the endometrial physiology during the estrus cycle and the first weeks of pregnancy. From a morphological point of view, the bovine endometrium consists of the juxtaposition of large glandular areas with smaller aglandular areas namely intercaruncular and caruncular regions respectively. These two regions are lined by a monostratified luminal epithelium (LE) with convex polygonal cells [1]. In glandular areas, the luminal epithelium penetrates deeply into the underlying tissue and constitutes the branched columnar glandular epithelium (GE). The underlying supporting stroma consists of fibroblastic stromal cells (ST) within a collagen-based connective matrix including vascular and lymphatic vessels as well as infiltrating immune cells. The endometrium is a hormonally regulated tissue and exhibits considerable functional changes during the estrus cycle, which are mainly regulated by progesterone (P4), oestrogens and oxytocin [2]. In bovine, early research using cDNA arrays to analyze changes in gene expression identified a large number of genes expressed differently during the estrus cycle [3, 4]. Others transcriptional profiling studies have also been performed to determine either the progesterone-regulated genes [5] or the conceptus-induced changes in gene expression [6]. Additionnaly, changes in the global transcriptome of bovine endometrium induced by exposure to blastocysts before and after the conceptus elongation [7] have been investigated to identify novel genes dependent and independent on IFNt [8]. Endometrial transcriptomic studies have been implemented in the context of embryo loss in cattle, which is a major cause of low reproductive efficiency and infertility. In heifers, differences in the endometrial transcriptome have been related to fertility classification [9, 10]. In almost all studies, transcriptomic profiling was performed on the endometrium as a whole without further refinement. In situ hybridization of transcripts or immunolocalization of proteins were often performed to identify the cell type expressing genes found to be differentially expressed. However, only a small number of transcripts or proteins have been the focus of localization studies. Some studies have attempted to gain a better understanding of the spatiotemporal changes in global gene expression. RNA-sequencing has been used to compare the transcriptome and ability of the ipsilateral and contralateral uterine horns to support preimplantation conceptus survival [11]. Similarly, locoregional differences in the endometrial transcriptome have been demonstrated between the caruncles and intercaruncles in early pregnancy [12] suggesting that the transcriptome should be thoroughly investigated according to the cell types and structures of the endometrium. Previous studies in the mouse indicated cell type-specific differences in transcriptome changes between luminal and glandular epithelium in early pregnancy [13]. Other studies using laser capture microdissection (LCM) to isolate different endometrial cell types have demonstrated their hight potential for understanding the differences in the transcriptional events occurring during the estrus cycle and early pregnancy in ovine [14] and porcine endometrium [15]. To our knowledge, no equivalent study has been performed on the bovine endometrium. The aim of the present study was to characterize the transcriptome using RNA-sequencing in the three endometrial cell types (LE, GE and ST) isolated by LCM at day-15 of the estrus cycle. Since previous in vivo studies showed the effects of metabolic imbalance on endometrial gene expression during the early postpartum period [16], the specific impacts of post-partum negative energy balance on the three endometrial cell-types were also investigated and results reported in a companion paper (under review [17]).

## Results

**Fig. 1:**
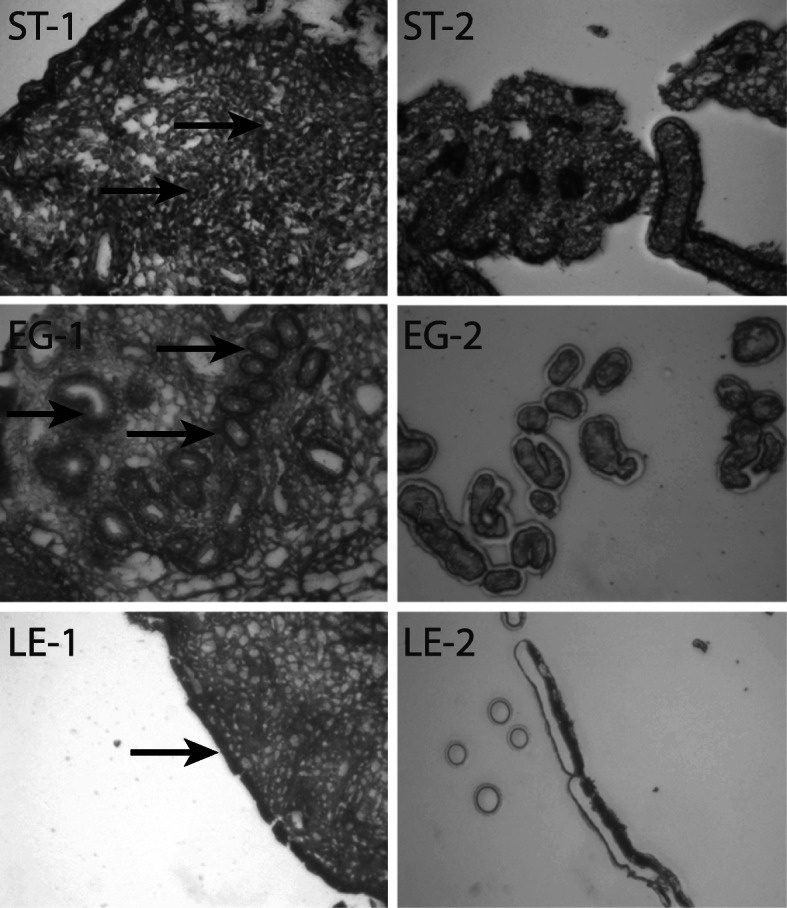
Isolation of the three bovine endometrial cell types by LCM: stromal cells (ST), glandular epithelial cells (GE) and luminal epithelial cells (LE), before [(1): left column and arrows)] and after [(2): right column] capture by LCM. (400x magnification)

All cows were cyclic (one full cycle or more) before initiation of the synchronization treatment. Commencement of luteal activity determined as day of first progesterone value above threshold of 3 ng/ml was 22.8 ± 10.3 days; mean ± SD). Milk progesterone concentrations during the days before biopsy sampling are shown in the supplementary figure-S1. All cows presented high progesterone concentrations (mean ± SE; 9.88 ± 2.12 ng/mL) and were in the luteal phase at time of endometrial biopsy. Depending on cell type, as many as 30 slides of endometrial sections per animal biopsies had to be processed using LCM to obtain the amount of RNA required for RNA sequencing (Table S1). RNA integrity was preserved during the isolation process and ranged from 7.23 to 7.75. Figure 1 evidences the cell capture for the GE, LE and ST cells. The absence of contamination of samples by infiltrating immune cells was verified from the screening for immune cell-specific markers in the lists of genes corresponding to each cell type from the RNAseq analysis. None of these specific markers from immune cells were detected at levels such as they could possibly interfere with the expression profiles of endometrial cells (Table S2).

### RNA-sequencing of cell type-specific samples collected by LCM

**Fig. 2:**
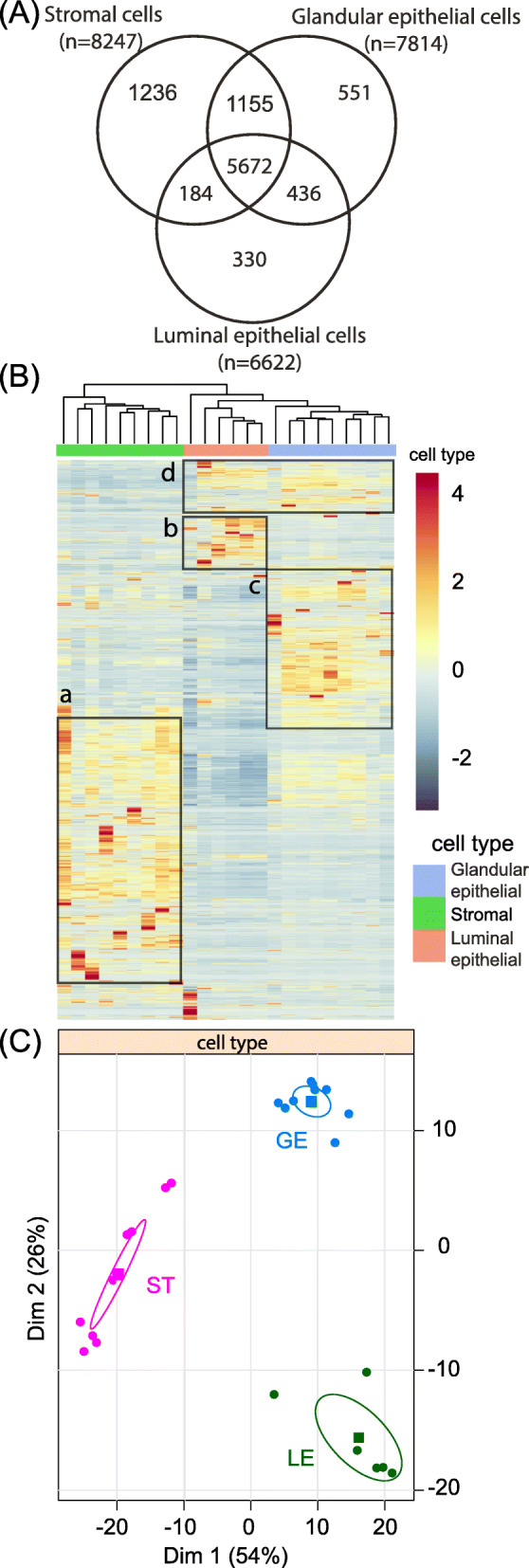
Transcriptomic analysis of endometrial cell types. (A) Venn diagram from genes expressed more than 10 TPM in specific endometrial cells (LE, luminal epithelial cells; GE, glandular epithelial cells; ST, stromal cells) (numbers of identified genes are indicated). (B) Heat map of genes expressed by ST, GE and LE cells and clustering of the three cellular types (the colors show the relative level of expression. Boxes highlight the more expressed genes for each cell type [(a): stromal cells; (d): luminal epithelial cell type; (c): glandular epithelial cells: (d): epithelial cell type]. (C) Principal component analysis for clustering expressed genes of the three endometrial cell types. Confidence ellipses around the barycenter of each cell type are shown

**Fig. 3:**
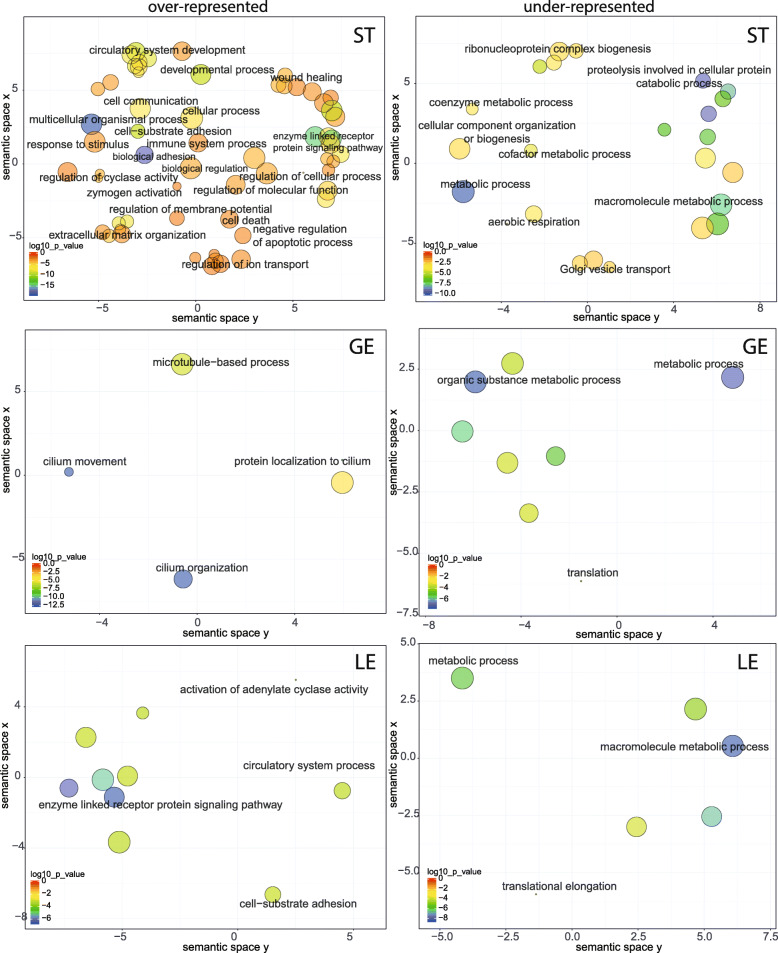
Scatterplot representation of biological process GO terms in semantic space using REVIGO. GO terms overrepresented in the list of genes specific to the three different cell-types of bovine endometrium (ST: stromal cells; GE: glandular epithelial cells; LE: luminal epithelial cells). Each circle corresponds to log 10 *p*-values according to the color scale shown at the bottom left of each figure. The size of each circle is proportional to the size of GO terms

The sequencing depth of RNA-seq libraries was in the range of 60 to 100 million reads per sample for each endometrial cell type. A total of 22,915 transcripts with a unique Identifier were found. Salmon’s method provides both read counts and TPM (transcripts per million), and the latter expression is more appropriate when comparing relative abundance between different cell types or tissues [18]. Before comparing the differences in gene expression between the endometrial cell types, transcripts whose average value, computed from biological replicates, were less than 10 TPM were regarded as biological background noise, partly independent of transcription regulation and discarded. The number of expressed genes detected (higher than 10 TPM) was 6622, 7814 and 8242 for luminal epithelial cells (LE), glandular epithelium (GE) and stromal cells (ST), respectively (Fig. 2A). In the RNA-Seq analysis, the highest number of detectable expressed genes (8242) in the LCM datasets was obtained for ST and the lowest number of detectable genes (6622) was observed for LE. As displayed on the Venn diagram (Fig. 2A), 5672 genes were expressed by all three cell types. A total of 1236 genes were expressed exclusively by ST cells, which represents 15% of all genes expressed by this type of cell, while only 551 (7% of all genes expressed) transcripts were specific to the GE cells and 330 (5%) transcripts specific to LE cell. The lists of genes specifically expressed by each cell type are provided in additional file (TableS3_LE_GE_ST.xlsx). An overview of the GO terms associated to genes specifically expressed by each cellular type is visualized in Fig. 3. The list of 5672 genes expressed in common between the three cell types was used as a reference list for PANTHER overrepresentation tests. Over and under-represented GO terms for biological process were visualized using REVIGO algorithm to reduce term redundancy (corresponding tables of GO terms are provided in additional file (TableS4_GO-REVIGO.xlsx). Respectively 97, 14 and 13 clusters of GO terms were over-represented in ST, GE and LE cells whereas 45, 11 and 8 were under-represented. Numerous metabolic processes were under-represented in the three lists of genes specifically expressed by each cell type which means that most of the genes involved in metabolism are shared ones. For ST, over-represented biological processes included many regulation processes and response to stimulus, cell communication and cell adhesion, extracellular matrix organization as well as developmental process and wound healing. For GE, cilium organization, cilium movement, protein localization to cilium and microtubule-based process were the only four main biological processes enriched. For LE, over-represented biological processes were enzyme linked receptor protein signaling pathway, cell-substrate adhesion, circulatory system process and activation of adenylate cyclase activity.

Heatmap (Fig. 2B) illustrates that the hierarchical clustering obtained from gene expression unambiguously regroups samples of each cell-type. The most expressed genes for each cell type are highlighted and framed by boxes (Fig. 2B). The corresponding statistical analyses revealed that 8070 genes were differentially expressed (adjusted *p* value < 0.05) between GE and LE cells (3921 genes greater expressed in GE vs. 4149 in LE). The expression of 10,258 genes differs between ST and LE (5319 genes more expressed in ST vs. 4939 more expressed in LE). The level of expression of 9541 genes differs between GE and ST (4538 genes more expressed in GE vs. 5003 in ST).

The principal component analysis confirms the clear separation of the samples from the three cell types (Fig. 2C). The first two dimensions explain 80% of the variability. The first dimension distinguishes epithelial cells from ST whereas the variation associated to the second dimension relates to differences of expression between GE and LE cells. Supplementary table (Table_S5_PCA.xlsx; sheets 1 and 2 for the first dimension, sheets 3 and 4 for the second dimension) show the most characteristic genes according to each dimension (at *p* < 0.01); 124 genes are associated with ST while 217 are associated with the two groups of epithelial cells. Looking at dimension 2, 113 genes are related to GE while 83 genes are attached to LE.

Genes negatively correlated to dimension-1 correspond to genes over-expressed in ST (Table_S5_PCA.xlsx; sheet 1). Among them, there is a significant over-representation of genes that are involved in extracellular matrix organization (R-BTA-1474244), in integrin signaling pathway (P00034) and in cadherin signaling pathway (P00012) (Table 1).

**Table 1:** Enrichment analysis using Statistical Overrepresentation Test (PANTHER 14.1) on genes negatively correlated to dimension-1 of PCA and associated to endometrial stroma cells (ST); GO terms are provided following PANTHER classification

Panther Classification	fold Enrichment	FDR	genes
PANTHER Pathways			
Cytoskeletal regulation by Rho GTPase (P00016)	9.84	1.09E-02	*ACTA2, MYH11, ACTG2, MYLK, TUBB6*
Integrin signaling pathway (P00034)	8.58	2.62E-04	*COL1A2, COL6A3, COL3A1, ACTA2, ARHGAP10, ACTG2, COL1A1, RND1, RND3*
Cadherin signaling pathway (P00012)	7.27	3.12E-02	*ACTA2, CDH11, CDH13, ACTG2, PCDH9*
PANTHER GO-Slim Biological Process			
circulatory system development (GO:0072359)	11.56	3.90E-02	*PDLIM3, SGCG, TNMD, MYLK, THBS1, APOLD1*
Reactome pathways			
Smooth Muscle Contraction (R-BTA-445355)	36.62	6.19E-02	*ACTA2, MYH11, TPM2*
Formation of Fibrin Clot (Clotting Cascade) (R-BTA-140877)	36.62	4.64E-02	*SERPINE2, SERPING1, F10*
ECM proteoglycans (R-BTA-3000178)	33.29	1.74E-02	*COL6A3, TNXB, COL1A1, DCN*
Extracellular matrix organization (R-BTA-1474244)	9.36	1.08E-02	*COL5A2, COL1A1, ADAMTS1, SMOC2, COL1A2, ECM2, MMP19, SULF1, COL3A1, MFAP4*

Other genes associated with smooth muscle contraction (R-BTA-445355), cytoskeletal regulation by Rho GTPase (P00016) and with formation of fibrin clot (R-BTA-140877) are also more expressed in ST.

Genes positively correlated to dimension-1 are over-represented in both GE and LE Table_S5_PCA.xlsx; sheet 2). Table 2 shows enrichment of genes coding for proteins involved in epithelial cell differentiation (GO:0030855), epithelium development (GO:0060429) and in cell adhesion (GO:0007155). This first group of genes encodes proteins for cell junction (PC00070) and tight junction (PC00214. A second group of genes associated and over-represented in the epithelial cells is encoding proteins for secondary carrier transporter (PC00258) and transporter (PC00227). By looking in more details (Table_S5_PCA.xlsx; sheet 2), epithelial cells are enriched in genes involved in cellular response to stimulus (GO: 0051715) and signal transduction (GO: 0007165) *(RHPN2*, *DYNCI1, RAB25*, *F2RL1, ITGB6, LPAR3, KSR2 and ERBB3).* Genes encoding proteins for catalytic activity (GO:0003824) such as enzymes of metabolism *GPT2, PLA2G4A, AKR1B* and *IDH1* are also over-expressed in these cells, as well as genes associated to EGF signaling pathway (P00018) and cell proliferation (*MAPK13*, *PEBP4*, *ERBB3*, *CCNA1* and *RAB25*).

**Table 2:** Enrichment analysis using Statistical Overrepresentation Test (PANTHER 14.1) on genes positively correlated to dimension-1 of PCA and associated to endometrial luminal and glandular epithelial cells (LE, GE); GO terms are provided following PANTHER classification

Panther Classification	fold Enrichment	FDR	genes
PANTHER GO biological process complete			
epithelial cell differentiation (GO:0030855)	5.59	1.39E-04	*CLDN3, SYNE4, LRP2, F2RL1,DLX6, ELF3, SPINT1, PHGDH, OVOL1, TACSTD2, ST14, EHF, MSX1, EPCAM, ST14, KDF1, IRF6, TJP3, HNF1B, SLC44A4, RAB25, DSP, MCOLN3*
epithelium development (GO:0060429)	3.87	2.05E-04	
cell adhesion (GO:0007155)	3.19	5.76E-02	*CLDN3, AZGP1, DSG2, PERP, FOLR1, FOLR1, SPAG6, EPCAM, CLDN7, CLDN4, CXADR, TJP3, IGSF5, CDH1, ITGB6, DSP*
PANTHER Protein Class			
tight junction (PC00214), cell junction protein (PC00070)	15.64	1.96E-03	*CLDN3, CLDN8, CLDN7, CLDN4, TJP3*
secondary carrier transporter (PC00258), transporter (PC00227)	8.55	2.11E-06	*SLC13A5, SLC44A3, SLC5A11, SLC5A11, SLC39A2, SLC27A6, SLC7A4, SLC5A1, SLC27A2, SLC34A2, SLC44A4, SLC5A8*
PANTHER Pathways			
Serine glycine biosynthesis (P02776)	44.23	1.65E-02	*PSAT1, PHGDH*

When addressing the genes associated to GE cells (Table_S5_PCA.xlsx; sheet 3) and according to Panther Molecular Function classification, 20 genes are related to catalytic activity (GO:0003824), 12 to binding (GO:0005488) and 8 to transporter activity (GO:0005215). According to Panther Biological Process classification, 20 are related to cellular process (GO:0009987), 16 to metabolic process (GO:0008152), 12 to biological regulation (GO:0065007), 9 to immune system process (GO:0002376) and 8 to response to stimulus (GO:0050896). Table 3 summarizes the over-representation of genes associated to GE cells. These genes encode proteins associated to related GO terms: microtubule (GO:0005874), intraciliary transport particle (GO:0030990), ciliary plasm (GO:0097014) and plasma membrane bounded cell projection cytoplasm (GO:0032838). These proteins are involved in axonemal dynein complex assembly (GO:0070286) and cilium movement (GO:0003341).

**Table 3:** Enrichment analysis using Statistical Overrepresentation Test (PANTHER 14.1) on genes positively correlated to dimension-2 of PCA and associated to endometrial glandular cells (GE); GO terms are provided following PANTHER classification

Panther Classification	fold Enrichment	FDR	genes
PANTHER GO-Slim Biological Process			
axonemal dynein complex assembly (GO:0070286)	56.29	3.94E-02	*CCDC65, ZBBX, DNAI1, DRC1, DRC3, DAW1, CFAP45, DNAH5, DNAH9*
cilium movement (GO:0003341)	26.63	4.93E-04	
PANTHER GO-Slim Cellular Component			
axoneme (GO:0005930)	15.58	2.16E-02	*CCDC65, SPAG17, DNAI1, DRC1, DNAH9*
ciliary plasm (GO:0097014)	15.01	1.97E-02	
plasma membrane bounded cell projection cytoplasm (GO:0032838)	13.76	2.25E-02	
intraciliary transport particle (GO:0030990)	7.94	3.54E-02	*CCDC65, FAM183A, SPAG17, ZBBX, DNAI1, DRC1, DNAH5, DNAH9*
microtubule (GO:0005874)	7.64	2.88E-02	
integral component of membrane (GO:0016021)	3.40	8.29E-2	*ADAMDEC1, LRP2, MGAT4C, CLDN10, CLDN8, CYP2D14, GJB5, LRAT, LDLRAD1, MCOLN2, MCOLN3, NXPE2, NRCAM, SDR16C5, SLC13A5, SLC15A2, SLC27A6, SLC36A2, SLC38A11, SUSD2, SV2B, TMPRSS2, TMEM144*
extracellular region (GO:0005576)	2.27	1.79E-02	*BPIFB1, SERPINE2, PLA2G10, MPTX, MGP, SPAG17, CPN1, PIP, SUSD2, S100B, CPN1, TDGF1, WIF1* *DNAI1, BPIFB1, AGT, MYOC, PIP, MYOC, IHH*

GE cells are also characterized by a significant number of genes coding proteins which are embedded in the cell membrane (integral component of membrane GO:0016021).

LE cells are mainly characterized by genes coding for enzymes metabolite interconversion enzyme, PC00262) and for transporter (PC00227) (Table_S5_PCA.xlsx; sheet 4). Panther overrepresentation test (Table 4) identifies enrichment for two groups of genes encoding transporters (secondary carrier transporter, PC00258) and proteases inhibitors (protease inhibitor, PC00191). Moreover, according to GO cellular component classification, there are also a large number of genes products that are known to be secreted from cells into the extracellular space (extracellular space, GO:0005615). LE expressed *PTGS2* and *OXTR* which are involved in oxytocin-stimulated prostaglandin release. Four LE genes *GPDL1, FST, NPPC* and *NR4A1* pointed out the gonadotropin-releasing hormone receptor pathway (P06664) in the endometrial luminal cells.

**Table 4:** Enrichment analysis using Statistical Overrepresentation Test (PANTHER 14.1) on genes negatively correlated to dimension-2 of PCA and associated to endometrial luminal cells (LE); GO terms are provided following PANTHER classification

Panther Classification	fold Enrichment	FDR	genes
PANTHER Protein Class			
secondary carrier transporter (PC00258)	11.91	5.28E-04	SLC4A11, SLC5A11, SLC39A2, GDA, SLC5A8, cationic amino acid transporter 3-like
protease inhibitor (PC00191)	11	4.38E-04	A2M, ITIH4, FST, IGFBP2, IGFBP1, AHSG, SLPI
GO cellular component complete			
extracellular space (GO:0005615)	3.55	5.38E-04	GPLD1, A2M, ITIH4, CHGA, FST, SFRP4, ULBP21, FAM3B, LCAT, CFB, TINAGL1, IGFBP2, FGF9, TNC, IGFBP1, TACSTD2, SCG3, NPPC, LAMB3, SMPDL3B, GPRC5A, AHSG, SLPI, PRSS22, FGFBP1

## Discussion

### Transcriptome of the three endometrial cell types

Our results fully confirm that stromal cells, glandular and luminal epithelial cells reveal specific molecular signatures as documented before in studies using LCM in human [19], sheep [14] and horse [20]. As the endometrial biopsies were all collected in the luteal phase of a synchronized estrous cycle, the conclusions of this study are relevant for the transcriptional events which are related to the endometrium exposed to high progesterone levels. Regulation of gene expression of the three endometrial cell types, under elevated estrogen concentrations during the follicular phase of the cycle would deserve similar studies*.* Our results based on biopsies collected in the luteal phase, have shown that a higher number of genes with a strong constitutive expression in stromal cells when compared with epithelial cells (either glandular or luminal) are different from the expression pattern observed at the beginning of pregnancy [20]. This may result from differences between species but could also reveal the changes induced by the conceptus on the endometrial transcriptome previously reported from full tissue [16, 21] and epithelial cells [14].

Using a cut-off of 10 TPM, different numbers of genes were expressed in the three endometrial cell types. ST expressed 5 and 25% more genes than GE and LE, respectively. However, as reported before from a large variety of tissues [22], and the three laser-dissected cell types of porcine endometrium [15], our results confirm that a high number of genes are expressed in common in different endometrial cell types. In the present study, 70 to 85% of genes were expressed in all cells suggesting either “house-keeping” functions or genes encoding proteins with functions common to the endometrium while lower proportions (5, 7 and 15% for LE, GE and ST, respectively) were restricted to each cell type indicating that they code for proteins supporting the functional specialized signature of each cell type. When compared to porcine endometrium [15], the number of genes showing cell-specific expression is in the same order of magnitude for GE and LE, but appears different for ST cells where this number is ten times higher. These differences in specific expression between cell types, especially the large number of functions enriched in ST are well reflected by the REVIGO analysis.

Regardless of the cut off chosen and related limitations, the above studies illustrated huge differences in gene expression patterns between cell types corresponding to specialized functions. This confirms that separating cell types is more appropriate and possibly less biased to decipher the impacts of any factor on a given tissue than former approaches based on full tissue. The clear clustering obtained when analysing the full transcriptome, indicates that luminal and glandular epithelial cells are more close to each other and differ from stromal cells. These similarities may reflect common functional properties and/or may be related to the common epithelial nature of these cells.

The RNaseq analysis generated 20,782 Ensembl stable identifiers. Among these mRNAs, 8070 (38%), 9541 (45%) and 10,258 (49%) were found differentially expressed between GE and LE, ST and LE, GE and ST respectively. As mentioned above, there are fewer differential expressed genes between GE and LE than between ST and the two epithelial cell types. As a common trend, The REVIGO analysis showed that genes encoding proteins related to metabolism were under-represented among genes specifically expressed by each cell type.

The stroma is dense and composed mainly of fibroblasts which produce the extracellular matrix. It contains also blood vessels which may be the source of endothelial cells in samples. In addition, depending on the physiological and health status of the cows, the stroma contains a variable number of resident and migrating immune cells such as macrophages, lymphocytes and eosinophils. However, Laser capture microdissection, by carefully selecting the cells to be picken, ensures that the sample is not contaminated by blood vessels. The presence of infiltrating immune cells may be a source of heterogeneity as these although visible, may be more difficult to eliminate from the stroma samples. The former selection of cows with low density of immune cells from cytology probably contributed to limit this possible source of contamination and we could not find tracks of specific RNA signatures of immune cells in our samples. Other sources of heterogeneity of stroma samples may exist which are more difficult to control. Most particularly, the stromal cells constituting the endometrial caruncle should have a different expression phenotype from that of the basal layer stromal cells which is located near the myometrium. Also, it has been suggested that the mammalian endometrium may contain adult stem cells of different types [23] which would be source of heterogeneity of the stromal cell population. The presence of these cells could confer additional RNA signatures and this could partly explain why more genes were differentially expressed in ST samples than in GE and LE samples.

An exhaustive analysis of the differentially expressed genes is beyond the scope of the discussion of this article and detailed information is provided in the supplementary tables. Below we have focused on highlighting the main genes that strongly contribute to the separation between the 3 cell types observed in PCA and for which expression may lead to the phenotype of each cell type. Using the official gene symbol approved by the HUGO Gene Nomenclature Committe, we performed a search of previously published articles limited to genes contributing to the dimensions of PCA. Search for terms [(endometrium or uterus) AND (bos or ovis or cow or bovine or ovine or sheep or ewe)], was combined in the PubMed Advanced Search Builder. Results indicate that only 10, 28 and 31% of the genes identified in the present study had been previously described in the uterus of ruminant species for respectively GE, LE and ST cells.

The genes associated with GE and LE, which distinguish the two types of epithelial cells from stromal cells, are all related to GO terms typical of epithelia (GO: 0030855, epithelial cell differentiation; GO: 0060429, epithelium development; PC00214: tight junction; PC00070: cell junction protein). *CDH1* was found expressed in GE and LE as previously described in the ovine uterus [24] whereas *CDH16* and *CDH17* was mentioned to be localized only in LE. *CDH1* is involved in organization of epithelium in mouse and its ablation causes the absence of endometrial glands [25]. Occludin (OCLN) which is an important protein for tight junction assembly that preserves the epithelial barrier function was expressed in both GE and LE. Temporal and spatial modifications in *OCLN* expression was reported to be related to cyclic and pregnant ewes [24]. LE expressed two additional cadherins, *CDH16 and CDH17,* which were never reported in uterus of any species. *CDH16* and *CDH17* are cadherins containing 7 cadherin domains and extensively shown to be expressed in the intestinal epithelial cells and kidney respectively [26]. On the contrary, ST cells express here two particular cadherins, *CDH11* and *CDH13* which have not, to our knowledge, been found in the uterus. CDH11 is a type II classical cadherin lacking cell adhesion recognition sequence and has been reported to be involved in the process of epithelial to mesenchymal transition [27]. CDH13 is an atypical member of the cadherin family, without transmembrane domain which has been associated with poorer prognosis in various cancers [28]. We report here also the expression of six different claudins (CLDNs) which label the three cell types differently. *CLDN3* are *CLDN7* are associated with GE and LE whereas *CLDN8* and *CLDN10* are expressed only by GE and *CLDN4* only by LE. *CLDN5* was associated here with ST but was previously detected in epithelial tissue in mouse uterus [29]. Consistently to what was reported in ewes [24] *CLDN3* and *CLDN4* were more expressed in LE and GE than in ST. However, contrary to the previous study [24], we were unable to detect *CLDN1* and *CLDN2* as junctional proteins differentiating cell types. This could be due to the fact that CLDN1, reported only at very low levels in LE and GE when progesterone reached its highest concentration [24], could not be detected in our experiment since the animals were biopsied on day 15 of the cycle. Alternatively, this may relate to differences between species. We identified a homeobox gene associated to GE and LE, *DLX6,* which has a similar pattern of expression in glandular and lining epithelial cells of mouse and human endometrium [30]. *EHF,* associated to GE and LE is an epithelium-specific transcription factor previously described in airway, intestinal and skin epithelia but never mentioned in the endometrium although it has been reported in uterine carcinosarcoma [31].

About 20 genes of membrane-bound solute carrier (SLC) transporter contribute significantly to the discrimination of the three cell types. Only one is specific to ST (*SLC2A3*), while all the others are associated to epithelial cells and reveal a significant enrichment of this class of proteins (PC00258). *SLC27A2, SLC34A2, SLC44A3, SLC44A4, SLC7A4, SLC22A16, SLC1A1* and *SLC23A1* are expressed by both GE and LE. Moreover additional solute carriers are specific either to GE or to LE. *SLC13A5*, *SLC15A2, SLC27A6, SLC28A3, SLC36A2 and SLC38A11* are associated with GE whereas *SLC4A11* and *SLC25A16* are associated with LE. Using a gene candidate approach, Bazer’s group described the regulated expression of 14 genes coding for SLC in the ovine endometrium [32–34]. The present exploratory study recognises only three of the SLC described in the above references. Our results for members of the SLC2 and SLC5 families, which are involved in sugar uptake, are partly in agreement with those previously published. We observed that *SLC5A1* was expressed in both LE and GE as in cow [35] and in ewe [34]. However *SLC5A11* was reported as more abundant in GE than in LE [34] whereas we found this gene specifically expressed in LE. Moreover we identify *SLC2A3* in ST whereas the type 4 of *SLC2A* was detected in these cells [35]. One may question the differences observed between the candidate approaches and our exploratory study. We focused on investigating the genes that differentiate cell types. By exploring the gene lists (TableS3_LE_GE_ST.xlsx) from RNAseq analysis, more than 100 SLCs are expressed in the bovine endometrium when using the cut-off of 10 TPM. When considering only those SLC markedly expressed (over 100 TPM), epithelial cells looks more enriched in SLC than stromal cells (14, 23 and 8 SLCs for GE, LE and ST respectively). This could be consistent with the fact that enrichment of receptors within the epithelium is essential for epithelial functions, particularly to regulate the composition of the luminal fluid [36].

GE are highly enriched for a large group of genes encoding for proteins related to cilium movement such as dyneins (GO:0003341). The dynein associated proteins are known to participate in numerous cellular functions involving microtubules bases movement [37]. Inside the cell, dynein powers the transport of membranes vesicles such as endosomes, lysosome, lipid droplets and vesicles. Predominantly expressed in ciliated cells, dynein drive the sliding of adjacent microtubules in the motile axonemes and provokes deformations resulting in cilia oscillations [37, 38]. For long, ciliated cells have been recognized as organelles involved in motility. In the bovine species, oviduct ovum transport is likely to result from ciliary beat [39]. Such a function remains difficult to warrant for the uterine glands. Motile cilia promote also fluid movement at the apical surface of the epithelium [38]. It could be speculated that the above role is certainly effective in the uterine glands as these are responsible for the secretion of the main part of the histotrophic molecules. However since the glandular epithelium of ruminant species is composed of fewer ciliated cells than non-ciliated cells [1, 40, 41], additional hypotheses are needed to explain the enrichment of GE with proteins of axonemal dynein complex assembly. On the contrary to mice where *IHH* was located in LE [42], we found this gene strongly associated with GE. In mice *Ihh* is suspected to be involved in uterine gland morphogenesis and epithelial cell proliferation. Since hedgehog signaling is known to be tightly coupled to the maintenance and function of primary cilia in various mammalian cell types [37], we suggest that both *IHH* and genes of the dynein complexes are involved in the maintenance of glandular epithelial cell functions in bovine.

The endometrium contains proteinase inhibitors and related molecules whose roles are not fully understood [43, 44]. Our results show that these proteinase inhibitors are mainly related to epithelial cells. Molecules such as *SERPINA14*, which is a major progesterone-induced protein secreted in the ruminant uterine fluid, is thought to act as an immune-modulator during pregnancy [43]. In our study, *SERPINA14* is associated with both GE and LE whereas the protein was previously reported to be preferentially located in GE [45]. *SERPINE2*, described as an endometrium plasminogen inhibitor in humans [46] and *SERPIN11* were not yet described in the ruminant uterus. ST also expressed two distinct serine-proteinase inhibitors, *SERPING1* and *SERPINE1.* AHSG, a plasma protein, produced primarily by the liver was previously reported in bovine endometrium [47]. In the current work, *AHSG* expression was associated with LE. AHSG is known to play a role in controlling the localisation and breakdown of IGFBP proteins within tissues. IGFBPs, which are involved in the bio-avaibility of IGFs, were intensively studied in the bovine uterus [48–50]. We found *IGFBP1*and *IGFBP2* as main markers of LE as described before [49, 51], whereas others have reported a widespread distribution in the endometrium [47]. Besides these well-known markers, LE expressed other genes, for which little is known in the bovine, encoding proteinase inhibitors involved either in the immune system, such as *SLPI* and *A2M* [52, 53], in the regulation of uterine glycocalyx (*ITIH4)* [54] or in the activin pathway controlling the development of conceptus (*FST)* [55].

Three ectonucleotide pyrophosphatases/phosphodiesterases; *ENPP3–5* were associated with both GE and LE. Surprisingly, *ENPP2* has not been described so far as a marker of epithelial cells although this enzyme has been shown responsible for the uterine synthesis of lysophosphatidic acid in ruminants [56, 57]. We identified *LPAR3* as an epithelial gene as previously reported [58] while genes coding for proteins involved in oxytocin-induced prostaglandins *PTGS2* and *OXTR* were only associated with LE. Our results are in agreement with previous studies showing that OXTR was exclusively observed in luminal epithelium during luteal phase in cycling cow [59].

By contrast, a very large number of functions including but not limited to, cell structure, angiogenesis, extra cellular matrix and immunity are enriched in ST whereas a lack of strong expression of these genes is observed in GE and LE. As awaited, among the genes most discriminating stromal cells, those involved in the production of extracellular matrix and collagen are highly represented. *COL1A2, COL3A1, COL7A1* and *COL3A3* encode proteins involved in dynamic remodeling of endometrial extracellular matrix and regulate embryo receptivity in cattle [60]. In addition, we identified here genes associated with extracellular matrix organization, which have not been previously described in the bovine endometrium. These includes *LOXL2*, responsible for the cross-linking of collagen and elastin [61], *ECM2* involved in the regulation of cell proliferation and differentiation [62] and *CRISPLD1* known to regulate extracellular matrix and branching morphogenesis [63]. These genes encode proteins that may have an important role in the formation of glands and vasculature of the bovine endometrium as well as *WT1,* already known to be preferentially expressed in stromal endometrial cells [64, 65].

We identify here also original genes related to stromal cell differentiation and cell migration such as *CDH11, PRELP, THY1* (the latter encoding a stem cell marker) [66], *GJA1* [67], *OSR2* [68], *P4HA3. PRLEP* gene expression has been reported to be regulated by the embryo in the bovine oviduct [69]. Contrary to the porcine endometrium where its expression was reported to be located in epithelial cells [[70], *NTRK2* was mainly expressed here in stromal cells. The expression of the NTRK2 gene, which encodes the receptor of brain derived neurotrophic factor, is conserved in mammalian uterus but its signaling function is not yet understood in the female reproductive system [71]. Genes known to be key regulators of uterine receptivity in different species such as, *HOXA10* and *HOXA11* belong also to the 50 genes top list characterizing ST as earlier shown in human [72], mice [73] and goat [74]. This list includes *CALPAIN7* [75] and *SNAI2* [76], involved in embryo attachment and implantation, and the disintegrins and metalloproteases *ADAMTS1* and *ADAM23* which encode key molecules for bovine endometrial remodelling [77]. In addition, a group of stromal genes including *SERPING1* [78], *C1R*, *C1S* [79], *SFRP1* and *IGF1* are involved in immune modulation of embryo maternal interactions and response to IFNs.

Finally, among these first 50 genes that best separate ST from epithelial cells, numerous ones have not been described so far in the mammalian endometrium. For instance, we could not find any information on the expression and function in the endometrium of the following genes and their encoded proteins: *MUSTN1, OSR2, TGM2, PCDH9, PGM5, MXRA5, MAMDC2, MRGPRF*, *RASD2, SULF1, RASL11A, ECM2, OLFML3* and *P4HA3*. These results may help to formulate new hypotheses for exploring new biological roles for genes of the stromal cells that are increasingly appearing as important cells in endometrial functions [80].

## Conclusion

The present study provides novel and specific information about gene expression in ST, LE and GE endometrial cells from postpartum dairy cows and illustrates specific signatures of these cells at day-15 of the estrous cycle. The most interesting result is that stromal cells express more genes than the two epithelial types and are associated with a greater number of pathways and ontology terms. The findings of this study will serve as a basis for in-depth investigations of cell type-specific molecular pathways and functions in bovine.

## Methods

### Animals and experimental design

This study was approved by the Uppsala Animal Experiment Ethics Board (application C329/12, PROLIFIC). After the study was conducted all cows have been kept in usual farm living conditions. Studies were conducted at the Swedish Livestock Research Centre in Lӧvsta, Uppsala, Sweden. The animals were kept in a loose housing barn with a voluntary milking system (VMS, DeLaval, Tumba, Sweden), and had free access to drinking water. Second lactation cows of the Swedish Red breed (SRB; *n* = 12) were fed two different diets i.e. i) high-energy diet (control, *n* = 6) targeting 35 kg energy-corrected milk (ECM) and ii) low-energy diet targeting (n = 6) 25 kg energy-corrected milk (ECM). Details about diets and relationships between diet, metabolic profiles and negative energy balance (NEB) were previously reported [81]. In complement to the present study, the effects of NEB profiles on gene expression in the three endometrial cell types were analyzed and are described in the companion paper [17]. All cows initially recruited in the experiment have been checked for uterine health by using both clinical examination, including ultra-sound examination and endometrial cytology. All cows included in further experiments (synchronization of estrus followed by uterine biopsies in view of LCM) had no clinical signs of uterine disease [82]. They presented less than 10% (four cows had percentages of immune cells between 7 and 10%, and all other cows presented less than 5% of immune cells counted from a total of 400 cells) of immune cells from endometrial cytobrush at 42–45 days post-partum, according to [83]. At day 60 after calving, estrous was synchronized using an intra-vaginal progesterone device (CIDR, Zoetis, Parsippany, NJ, USA) for a week followed by i.m. injection of 500 μg of prostaglandin analog (Estrumate®, MSD animal health, Madison, NJ, USA) intramuscular as described [84]. Fifteen days after visual estrus detection, endometrial tissue biopsies were collected under epidural anesthesia with 0.5 mg/kg of 1% lidocaine hydrochloride (1% Xylocaine®, Astra Zeneca, Cambridge, UK).

### Milk progesterone measurements and estrous cycle stage at time of biopsies

Whole milk samples were collected by the automatic milking machine, VMS (DeLaval) three times per week from Day 7 to Day 120 after calving. Milk progesterone concentrations were measured with a commercial enzyme-linked immunosorbent assay (ELISA) (Ridge way ‘M’ kit, Ridgeway Science, Gloucester, UK) as previously published [81]. The progesterone concentration profile was used to determine the estrous cycle stage at the time of biopsy sampling.

### Collection of endometrial biopsies

Endometrial biopsies were collected from the uterine horn ipsilateral to the corpus luteum by using Kevorkian-Younge uterine biopsy forceps (Alcyon, Paris, France). Biopsies were cut into 3 pieces (sizes ≈ 4 × 4 mm^2^). One of them was snap frozen in cold isopentane (2-Methylbutane, Sigma Aldrich, Saint Louis, MO, USA) previously placed in liquid nitrogen for 5 min, and immediately embedded in ≈1 cm^3^ optimal cutting temperature (OCT) compound (VWR, Radnor, PA, USA). OCT conditioned biopsies were then put into dry ice and kept at − 80 °C until sectioning. Tissue blocks were 8 μm sectioned with a cryostat (Leica CM1860 Cryostat, Wetzlar, Germany) at − 20 °C under RNA-free conditions. Tissue section slices were mounted on Super Frost slides RNA-free which were chilled on ice, following immersion in ice-cold 75% RNA-free ethanol and stored at − 80 °C until staining [85].

### Laser capture microdissection (LCM) and RNA isolation

All procedures used were those previously published [85]. Tissue sections were mounted on RNAse-free glass slides which were chilled on ice, following immersion in cold 75% RNA-free ethanol at − 20 °C in the cryostat and then transferred into 75% ethanol at RT (30 s), stained with 1% cresyl violet in ethanol (15 s), rinsed successively with 75% ethanol (30 s), 95% ethanol (2 × 1 min), and 100% ethanol (2 × 1 min) (anhydrous Ethanol absolute). Finally, the slides were completely dehydrated by immersion in pure xylene (M-xylene, Sigma-Aldrich, Saint-Quentin-Fallavier, France) for 2 × 5 min. Stained tissue sections were then immediately air dried. The LCM process was performed by using an ArcturusXT™ Laser Capture Microdissection System and software (Applied Biosystems®, Arcturus, ThermoFisher Scietific, Waltham, MA, USA), within 1 h to avoid RNA degradation. Luminal epithelial cells (LE), glandular epithelial cells (GE) and stromal cells (ST) were harvested in sufficient numbers to obtain at least 10 ng of total RNA for each endometrial cell type. Briefly, cells were captured from the slide onto LCM plastic caps (CapSure®Macro LCM Caps, Arcturus) by using infrared laser with the following settings: power range 75 to 90 mW, time 1300 to 3500 μsec and 200 mV intensity. Collected cells were then placed in a RNAse-free 0.5 mL microcentrifuge tube with 25 μL extraction buffer (provided together with the PicoPure™RNA isolation kit; KIT0202, Arcturus) and incubated for 30 min at 42 °C. Captured cells in PicoPure extraction buffer were frozen at − 80 °C before processing samples for RNA isolation. Total RNA from LCM samples was isolated and mRNA purified using the PicoPure™RNA isolation kit (KIT0202, Arcturus) following the manufacturer’s protocol. RNA integrity value (RIN values) and quantity were evaluated using the Pico RNA chip on the Agilent 2100 Bioanalyzer (Agilent technologies, Santa Clara, CA, USA). Mean RNA integrity (RIN) values obtained from LCM samples and from the full tissue samples issued from the same biopsy were similar (paired T-test; Table S1).

### RNA sequencing and data analysis

RNA sequencing libraries prepared from 24 samples were prepared and sequenced on GenomEast Platform (IGBMC, Cedex, France; http://genomeast.igbmc.fr/). Libraries were built using the Clontech SMART-Seq v4 Ultra Low Input RNA kit for Sequencing. Full length cDNA were generated from 4 ng of total RNA using Clontech SMART-Seq v4 Ultra Low Input RNA kit for Sequencing (Takara Bio Europe, Ozyme, Montigny-Le-Bretonneux, France) according to manufacturer’s instructions, with 10 cycles of PCR for cDNA amplification by Seq-Amp polymerase. Then, 600 pg of pre-amplified cDNA were then used as input for Tn5 transposon tagmentation using the Nextera XT DNA Library Preparation Kit (Illumina, San Diego, CA) followed by 12 cycles of library amplification. Following purification with Agencourt AMPure XP beads (Beckman-Coulter, Roissy, France), the size and concentration of libraries were assessed by capillary electrophoresis. Sequencing was performed on an Illumina HiSeq 4000 with 50 bp paired-end reads. Image analysis and base calling were performed using RTA 2.7.3 and bcl2fastq 2.17.1.14. Gene level exploratory analysis and differential expression were performed using the RNAseq workflow described by [86] and the update version http://bioconductor.org/packages/release/bioc/vignettes/DESeq2/inst/doc/DESeq2.html. html). The Salmon method [87] was used to quantify transcript abundance. The cDNA sequence database for *Bos taurus* was obtained from Ensembl (release-98; Bos_taurus.ARS-UCD1.2.cdna.all.fa) and was used to build a reference index for the bovine transcriptome (see details in [87]. After quantifying RNA-seq data, tximport method [88] (R package version 1.8.0) was used to import Salmon’s transcript-level quantifications to the downstream DESeq2 package (R package, version 1.20.0) for analysis of differential expressed genes (DEGs) with the statistical method proposed [89]. The package DESeq2 provides methods to test for differential expression by use of negative binomial generalized linear models using the design formula (~cell-type) to compare gene expression between endometrial cell-types. Principal component analysis was performed with DESeq2 and with FactoMineR (R package, version 1.4.1) using the variance stabilizing transformation output files from DESeq2. Heatmap was generated in R software using the pheatmap package (version 1.0.12) and Venn diagrams were plotted with VennDiagram package (1.6.20). DEGs were identified in comparison between cell types with an adjusted *p*-value of 0.05. The data have been deposited in NCBI’s Gene Expression Omnibus and are accessible through GEO Series accession number GSE169638 (https://www.ncbi.nlm.nih.gov/geo/query/acc.cgi?acc=GSE169638).

### Gene ontology and KEGG pathway analysis

Lists of genes expressed by the three types of endometrial cells were annotated into three categories of Gene Ontology (GO) pathways such as biological process (BP), cellular component (CC) and molecular function (MP) using PANTHER classification system (Protein Analysis THrough Evolutionary Relationships version 14.0, http://pantherdb.org). PANTHER overrepresentation tests were performed using all genes from the whole *Bos taurus* genome or from specified list. Lists of GO terms were summarized and visualized in semantic space by REVIGO (http://revigo.irb.hr/) [90]. The SimRel semantic similarity score was used and the threshold was set at 0.15.

### Statistical analysis

The results of milk progesterone concentration are presented as LSmeans ± S.E.M. Differences with associated *p*-value < 0.05 were considered to be significant.

## Supplementary Information

**Additional file 1: Figure S1.** Milk progesterone concentrations (ng/ml) following estrus synchronization of cows (*n* = 12) with CIDR 60 days after calving. All the endometrial biopsies were taken at Day 14–15 following visual oestrus detection and were performed during the luteal phase of the cycle. Progesterone values are expressed as mean +/− sem.

**Additional file 2: Table S1.** Number of samples of each cell type. RNA Integrity Number (RIN)] [mean value (± s.e.m)] and average number of tissue sections required to obtain at least 10 ng of total RNA in each endometrial cell type.

**Additional file 3: Table S2.** Expression of genes of key markers for immune cells in the three different endometrial cell types.

**Additional file 4: Table S3.** List of genes specifically expressed by the three endometrial cell types.

**Additional file 5: Table S4.** List of GO terms for under and over expressed genes three endometrial cell types.

**Additional file 6: Table S5.** List of genes expressed by endometrial cells according to the first two dimensions of the Principal Component Analysis.

## Data Availability

The data discussed in this publication have been deposited in NCBI’s Gene Expression Omnibus [91] and are accessible through GEO Series accession number GSE169638 (https://www.ncbi.nlm.nih.gov/geo/query/acc.cgi?acc=GSE169638). The gene accession numbers indicated in the additional files refer to the corresponding Ensembl identifier (https://www.ensembl.org/Bos_taurus/)(*Bos taurus*:ARS-UCD1.2). Gene names were retrieved using the Ensembl BioMart tool.

## Abbreviations

CIDR: Controlled Internal Drug Release
DAVID: Database for annotation, visualization and integrated discovery
DEG: Differentially expressed gene
Elisa: enzyme-linked immunosorbent assay
FDR: False discovery rate
GE: Glandular epithelial cell
GO: Gene ontology
KEGG: Kyoto encyclopedia of genes and genomes
LCM: Laser capture microdissection
LE: Luminal epithelial cell
NorFor: Nordic Feed Evaluation System
OCT: Optimal cutting temperature coumpound
PANTHER: Protein analysis through evolutionary relationships
PCA: Principal Component Analysis
PGE2: Prostaglandin-E2
PGF2α: Prostaglandin-F2α
REVIGO: Reduce, visualize gene ontology
RNA: Ribonucleic acid
RNA-Seq: RNA sequencing
SRB: Swedish Red breed
ST: Stromal cell
STRING: Search Tool for the Retrieval of Interacting Genes/Proteins
TPM: Transcripts per million
