# Supplementary figures and images for: Analysis of the transcriptome of bovine endometrial cells isolated by laser micro-dissection (1): specific signatures of stromal, glandular and luminal epithelial cells
Source: BMC Genomics. 2021 Jun 18;22:451. doi: 10.1186/s12864-021-07712-0 (PMC8212485; doi:10.1186/s12864-021-07712-0)

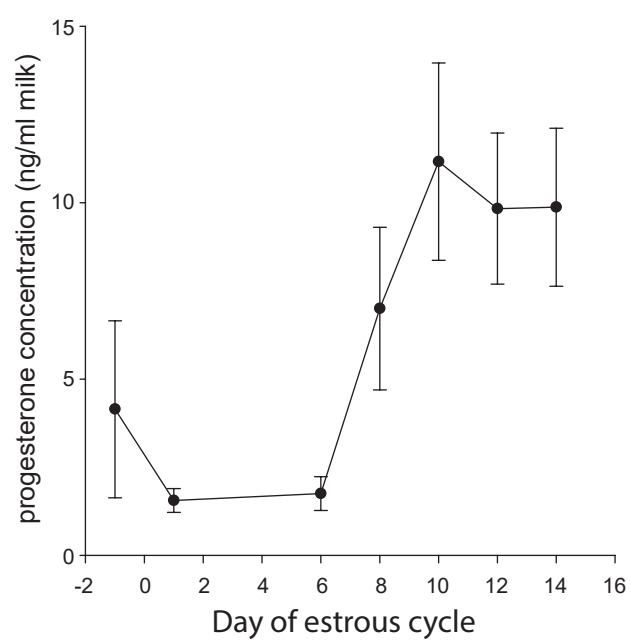

Figure S1

Supplement: Supplementary file 1 — Additional file 1: Figure S1. Milk progesterone concentrations (ng/ml) following estrus synchronization of cows (n = 12) with CIDR 60 days after calving. All the endometrial biopsies were taken at Day 14–15 following visual oestrus detection and were performed during the luteal phase of the cycle. Progesterone values are expressed as mean +/− sem. [file 12864_2021_7712_MOESM1_ESM.pdf]
