# Supplementary material for: Analysis of the transcriptome of bovine endometrial cells isolated by laser micro-dissection (1): specific signatures of stromal, glandular and luminal epithelial cells
Source: BMC Genomics. 2021 Jun 18;22:451. doi: 10.1186/s12864-021-07712-0 (PMC8212485; doi:10.1186/s12864-021-07712-0)
Supplement: Supplementary file 2 — Additional file 2: Table S1. Number of samples of each cell type. RNA Integrity Number (RIN)] [mean value (± s.e.m)] and average number of tissue sections required to obtain at least 10 ng of total RNA in each endometrial cell type. [file 12864_2021_7712_MOESM2_ESM.docx]

| endometrial cell types | number of samples | RIN | number of tissue sections  (for 10 ng of total RNA) |
| --- | --- | --- | --- |
| Full tissue section | - | 7.39 ± 0.13 | 1 ± 0 |
| Stromal cell (ST) | 9 | 7.23 ± 0.13 | 9 ± 3 |
| Glandular epithelial cell (GE) | 9 | 7.44 ± 0.13 | 13 ± 3 |
| Luminal epithelial cell (LE) | 6 | 7.75 ± 0.15 | 35 ± 3 |

Table S1: Number of samples of each cell type. RNA Integrity Number (RIN)] [mean value (± s.e.m)] and average number of tissue sections required to obtain at least 10 ng of total RNA in each endometrial cell type.
