# Supplementary material for: Analysis of the transcriptome of bovine endometrial cells isolated by laser micro-dissection (1): specific signatures of stromal, glandular and luminal epithelial cells
Source: BMC Genomics. 2021 Jun 18;22:451. doi: 10.1186/s12864-021-07712-0 (PMC8212485; doi:10.1186/s12864-021-07712-0)
Supplement: Supplementary file 3 — Additional file 3: Table S2. Expression of genes of key markers for immune cells in the three different endometrial cell types. [file 12864_2021_7712_MOESM3_ESM.docx]

| **Key markers for** | **Gene Name** | **ID Ensembl** | **Stromal Cell** | **Glandular Cell** | **Luminal cell** |
| --- | --- | --- | --- | --- | --- |
| LT, LB | CD8a | ENSBTAG00000021141 | nd | nd | nd |
| LT, LB | CD4 | ENSBTAG00000003255 | nd | nd | nd |
| LT, LB | CD3g | ENSBTAG00000006453 | 14 | 14 | nd |
| LT | CD2 | ENSBTAG00000017256 | nd | nd | nd |
| LT | CD3D | ENSBTAG00000006452 | 14 | nd | 14 |
| LB | CD19 | ENSBTAG00000032122 | nd | nd | nd |
| LT, NK | CD8b | ENSBTAG00000008956 | 34 | nd | nd |
| NK, LT, LB | CD11b | ENSBTAG00000047238 | nd | nd | nd |
| M | CD163 | ENSBTAG00000019669 | nd | nd | nd |
| M | CD68 | ENSBTAG00000000133 | 29 | nd | nd |
| M | CD86 | ENSBTAG00000013118 | nd | nd | nd |
| M | CD14 | ENSBTAG00000015032 | 36 | nd | 16 |
|  |  |  |  |  |  |
| LT, LB: lymphocytes T, B; NK : natural killer; M: macrophage | | |  |  |  |
| nd : non detected <10 TPM | |  |  |  |  |
| values in TPM (transcrit per million) | | |  |  |  |

TableS2: Expression of genes of key markers for immune cells in the three different endometrial cell types.
